# Supplementary material for: Transcriptomic Profiling Uncovers Molecular Basis for Sugar and Acid Metabolism in Two Pomegranate (Punica granatum) Varieties
Source: Foods. 2025 May 15;14(10):1755. doi: 10.3390/foods14101755 (PMC12111560; doi:10.3390/foods14101755)
Supplement: Supplementary file 1 [file foods-14-01755-s001.zip › supplementary Figures S1-S4.pdf]

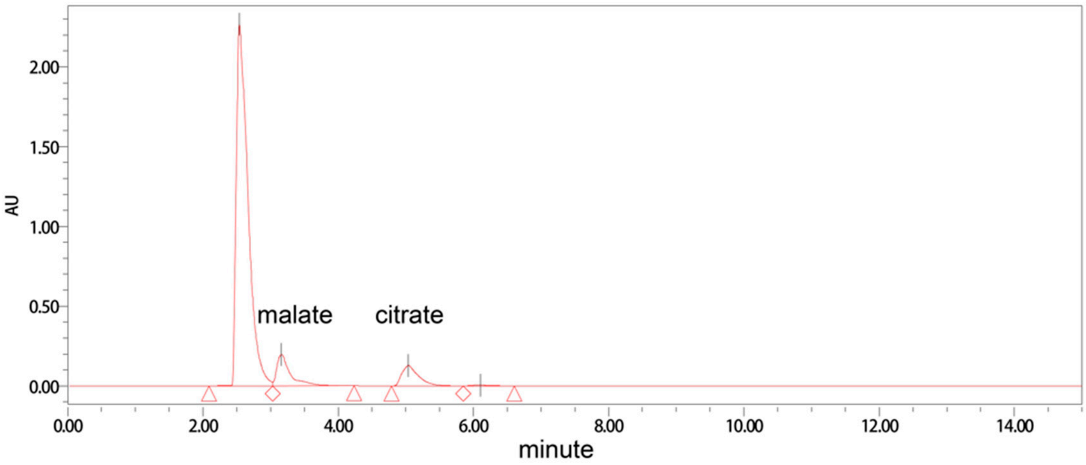

Figure S1. HPLC chromatogram of organic acid mixture standard sample

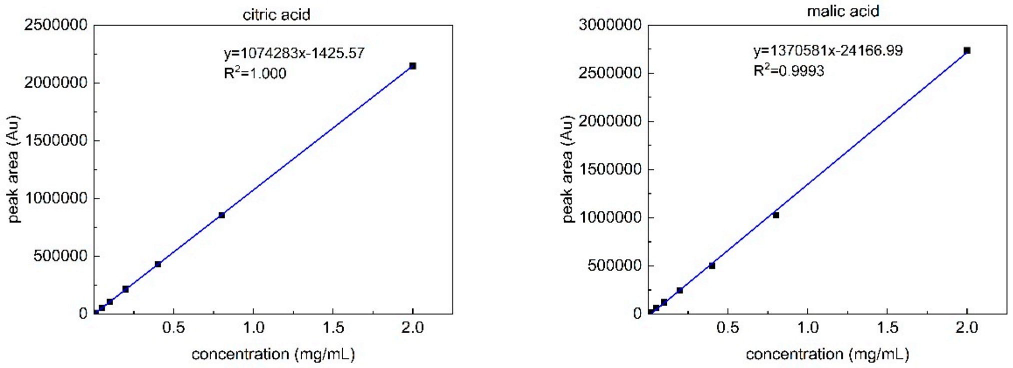

Figure S2, Standard curve of citric acid and malic acid component

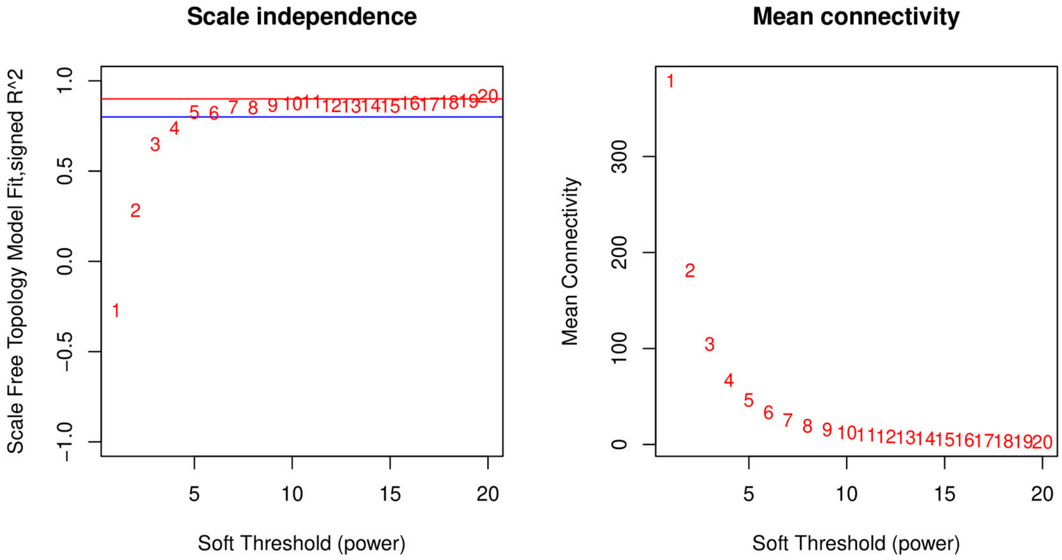

Figure S3. Analysis of the network topology for various soft-thresholding (β value) powers. The red dotted line is drawn at 0.9.

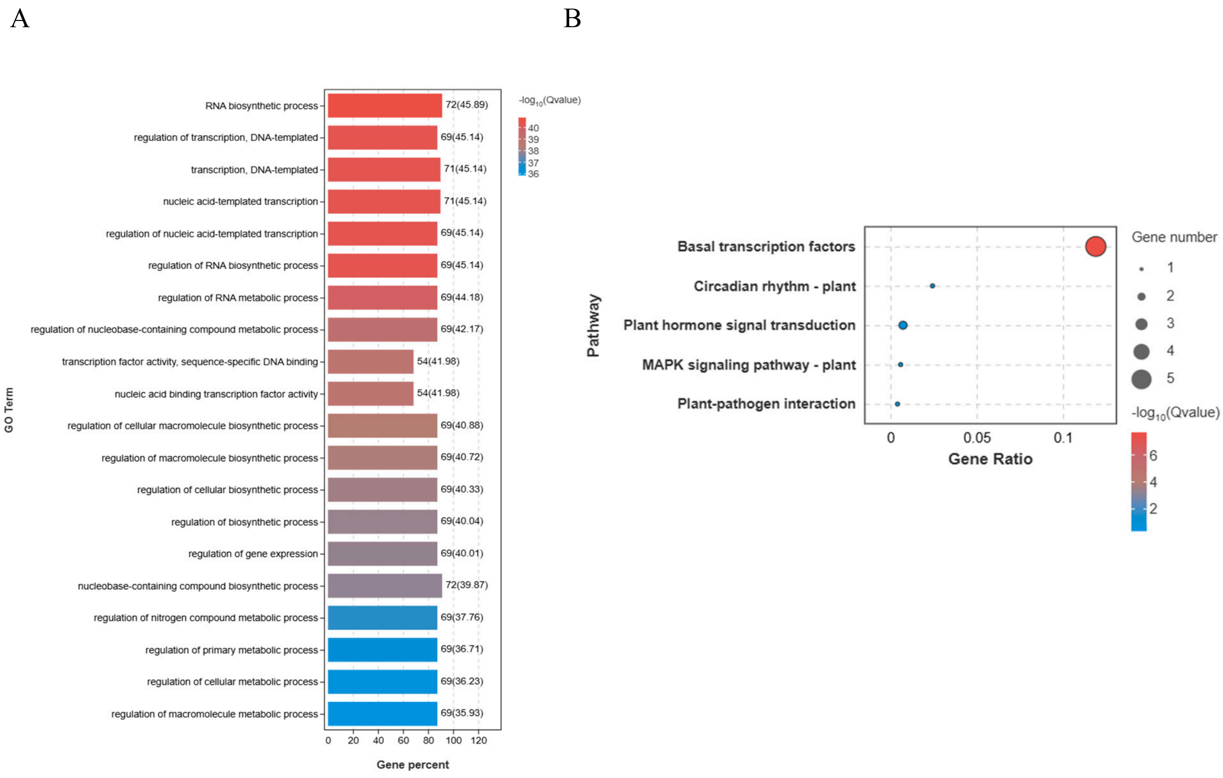

Figure S4. Enrichment of 129 TFs in green module. A.GO term of 129 TFs in green module. B.KEGG enrichment of 129 TFs in green module.
